# Integrative analysis of protein-coding and non-coding RNAs identifies clinically relevant subtypes of clear cell renal cell carcinoma

**DOI:** 10.18632/oncotarget.12340

**Published:** 2016-09-29

**Authors:** Zongcheng Li, Yaowen Chen, Shuofeng Hu, Jian Zhang, Jiaqi Wu, Wu Ren, Ningsheng Shao, Xiaomin Ying

**Affiliations:** ^1^ Beijing Institute of Basic Medical Sciences, Beijing 100850, China; ^2^ Translational Medicine Center of Stem Cells, 307-Ivy Translational Medicine Center, Laboratory of Oncology, Affiliated Hospital, Academy of Military Medical Sciences, Beijing 100071, China; ^3^ Department of Obstetrics and Gynecology, Fuzhou General Hospital of Nanjing Military Command, Fuzhou, Fujian 350025, China; ^4^ Department of Gastrointestinal Surgery, The First Affiliated Hospital of Jilin University, Changchun 130021, China

**Keywords:** subtyping, integrative analysis, transcriptome, ccRCC, non-coding RNAs

## Abstract

Protein-coding genes and non-coding RNAs cooperate mutually in cells. Integrative analysis of protein-coding and non-coding RNAs may facilitate characterizing tumor heterogeneity. We introduced integrated consensus clustering (ICC) method to integrate mRNA, miRNA and lncRNA expression profiles of 431 primary clear cell renal cell carcinomas (ccRCCs). We identified one RCC subgroup easily misdiagnosed as ccRCC in clinic and four robust ccRCC subtypes associated with distinct clinicopathologic and molecular features. In subtype R1, AMPK signaling pathway is significantly upregulated, which may improve the oncologic-metabolic shift and partially account for its best prognosis. Subtype R2 has more chromosomal abnormities, higher expression of cell cycle genes and less expression of genes in various metabolism pathways, which may explain its more aggressive characteristic and the worst prognosis. Moreover, much more miRNAs and lncRNAs are significantly upregulated in R2 and R4 respectively, suggesting more important roles of miRNAs in R2 and lncRNAs in R4. Triple-color co-expression network analysis identified 28 differentially expressed modules, indicating the importance of cooperative regulation of mRNAs, miRNAs and lncRNAs in ccRCC. This study establishes an integrated transcriptomic classification which may contribute to understanding the heterogeneity and implicating the treatment of ccRCC.

## INTRODUCTION

Renal cell carcinoma (RCC) is a serious human disease and accounted for nearly 61,000 (~3.7%) estimated new cancer cases and 14,000 (~2.4%) premature deaths in the United States in 2015 [1]. RCC can be subclassified into several major subtypes, including clear cell RCC (ccRCC, 70-80%), papillary RCC (pRCC, 10-15%), chromophobe RCC (chRCC, 3-5%) and renal oncocytoma (RO), each possessing distinct histological features and genetic characteristics and arising from different parts of the nephron [2–6]. It is believed that ccRCC and pRCC arise in the proximal tissue of the kidney whereas chRCC and RO originate from distal regions of the kidney [6, 7].

Despite the common origin of proximal tubules, ccRCC, the most common RCC subtype, is not a single entity but a collection of heterogeneous tumors. Clinically, tumor pathologic stages and histologic grades can be used to stratify patients, infer prognosis and guide treatment [8]. However, due to intra- and inter-tumoral heterogeneity [9, 10], stages and grades fail to explain molecular diversity in tumors and cannot satisfy requirements of precision therapy. So understanding the inherent molecular basis of these heterogeneities in ccRCC remains important and challenging.

Currently, some molecular characteristics of ccRCC have been uncovered, such as recurrent mutations of von Hippel-Lindau (*VHL*), *PBRM1*, *BAP1* and *SETD2* [11–14], and loss of chromosome 3p [11]. These genomic aberrations can potentially change the landscape of tumor transcriptomes by altering expression of global gene sets. For example, *VHL* mutations lead to imbalances of hypoxia inducible factors (*HIF-1α* and *HIF-2α*, or *HIF1A* and *EPAS1*) [15–17] and then dysregulation of cellular metabolism. Mutations in chromatin remodeling genes, including *PBRM1*, *BAP1* and *SETD2*, may affect additional functional pathways through chromatin remodeling/histone methylation [12–14]. Metabolism shift and epigenetic reprogramming are critical for the development and progression of ccRCC [11]. Notably, noncoding RNAs, including miRNAs and long non-coding RNAs (lncRNAs), can be important epigenetic regulators of various biological processes through transcriptional and post-transcriptional processing and even chromatin remodeling. Recent evidences show critical roles for noncoding RNAs in tumorigenesis and some have been reported to have regulatory functions in crucial pathways in ccRCC such as miR-21, miR-92 and miR-210 [11, 18–20].

Transcriptome data, including mRNA, miRNA and long non-coding RNA (lncRNA) expression profiles, have been utilized individually for molecular subtyping of ccRCC [11, 21, 22]. Brannon and colleagues used gene expression microarray data to identify two robust expression subtypes (ccA and ccB) with distinct gene expression patterns and divergent biological pathway [21]. The Cancer Genome Atlas (TCGA) Research Network established four stable mRNA and four stable miRNA subgroups that correlated with survival [11]. Malouf's group identified four subclasses of ccRCC with distinct clinicopathological and genomic features based on lncRNA expression profiles [22]. These studies show that each transcriptome type of mRNAs, miRNAs and lncRNAs contributes much to ccRCC subtyping.

Actually, mRNAs, miRNAs and lncRNAs cooperate reciprocally and form regulation networks in tumor cells. Expression profiles of each data type reflect intrinsic molecular characteristics of tumors from a single perspective and contribute partially to tumor classification. Therefore, integrative analysis of multiple transcriptome data types including protein-coding genes and non-coding RNAs may facilitate capturing the heterogeneous nature of tumors and these data can be used to identify concordant tumor subtypes [23].

In this study, we introduced an integrative analytical method, termed integrated consensus clustering (ICC), to establish a classification based on multiple transcriptome data types. We then applied ICC to ccRCC with matched mRNA, miRNA and lncRNA expression data sets from TCGA. We identified one RCC subgroup easily misdiagnosed as ccRCC in clinic and four robust ccRCC subtypes associated with distinct clinicopathologic and molecular features. Furthermore, we constructed a triple-color co-expression network and identified functional modules differentially expressed among subtypes and associated with prognosis. Our results suggest that integrative analysis of protein-coding and non-coding RNAs can effectively classify ccRCCs into subtypes with significantly different biological pathways and regulation mechanisms.

## RESULTS

### Integrated consensus clustering (ICC)

**Figure 1:**
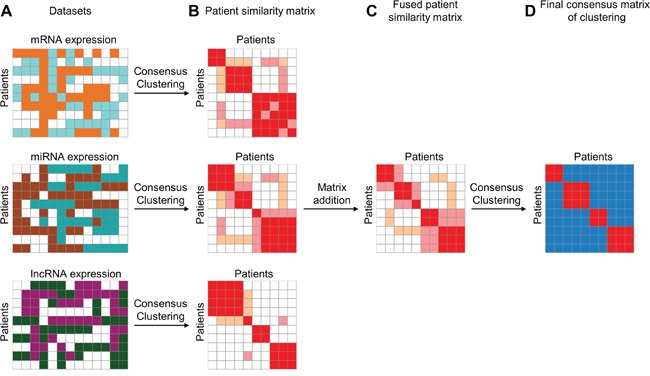
Illustrative steps of Integrated Consensus Clustering **A.** Three matrixes represent mRNA, miRNA and lncRNA expression profiles, respectively, for the same cohort of patients. **B.** Patient similarity matrixes are represented by the consensus matrix produced using consensus clustering algorithm for each data type independently. **C.** Fused patient similarity matrix is generated by matrix summation of three patient similarity matrixes derived from three different datasets. **D.** Final consensus matrix is produced by using consensus clustering on fused patient similarity matrix. The final consensus matrix can be used for evaluating clustering stability of ICC.

To integrate information from different data sources, we proposed ICC of three steps: (1) constructing a patient similarity matrix (PSM) from each data source; (2) integrating multiple PSMs into one fused PSM (fPSM); (3) obtaining a final clustering result (Figure 1). First, the consensus matrix of patients clustering was generated using the Consensus Clustering algorithm [24] for each data set. Each element of the consensus matrix represented the proportion of the corresponding two patients classified into the same cluster in multiple iterative clustering. We referred to the consensus matrices as PSMs, which could mask differences of diverse platforms and hence made PSMs comparable across various data sources. Next, PSMs were merged into an fPSM. Each element of fPSM was calculated as the sum of the corresponding elements from different PSMs and represented fused patient similarity. In the third step, the final consensus matrix (FCM) and clustering result were obtained from consensus clustering on fPSM.

To validate the feasibility of ICC, we applied ICC to three cancer cohorts from TCGA, namely BRCA (Breast invasive carcinoma), LGG (Brain Lower Grade Glioma), and LUAD (Lung adenocarcinoma) (Supplementary Table S1). Proportional area change under CDF was used for selection of the optimal K (Supplementary Figure S1A, S1C, S1E). After identification of stable clusters for each cancer type, overall survival analysis was performed. The results show that the overall survivals are significantly different among the subtypes in each of the three cancers (Supplementary Figure S1B, S1D, S1F). These results demonstrate that our ICC method can identify cancer subtypes with significant clinical relevance.

### Identification of stable clusters in ccRCC by integrated transcriptomic analysis

**Figure 2:**
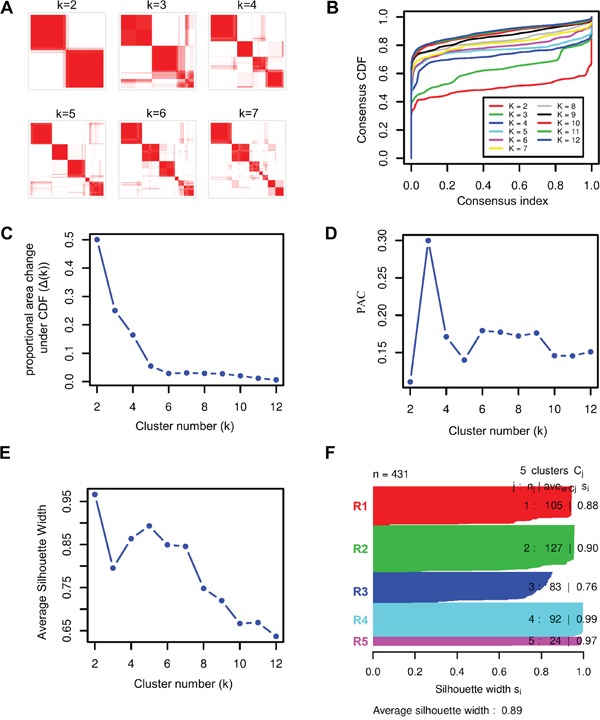
Identification of stable clusters of clear cell Renal Cell Carcinoma by using ICC **A.** Consensus matrixes of 431 TCGA ccRCC samples for each k (k=2 to 7), displaying the clustering stability using 1000 iterations of hierarchical clustering. **B.** Plot of cumulative distribution function (CDF) from consensus matrix for each k (k= 2 to 12). **C.** The Δ(k) vs k plot, indicating the optimal cluster number is k=5 where the ‘elbow’ occurs. **D.** Proportion of Ambiguous Clustering (PAC) vs k plot, allowing inference of the optimal k by the lowest PAC, that is 2 or 5. **E.** Average silhouette width vs k plot, showing the optimal cluster number k= 5 with respect to the major increase from k=3 to k=5 and the subsequent drop-off. **F.** Silhouette width profile calculated from the consensus matrix when k=5, which was selected as the optimal k.

We applied ICC to 431 primary ccRCC tumors with matched mRNA, miRNA, and lncRNA expression profiles from TCGA KIRC cohort to classify patients into clusters. To select the optimal cluster number, we assessed clustering stability using the FCM produced by ICC from k=2 to 12. For each cluster number k, an FCM was produced (Figure 2A) and empirical cumulative distribution (CDF) of each FCM was calculated (Figure 2B). To avoid subjective judgments, the optimal cluster number was determined by a combination of proportional area change under CDF (Δ(K), Figure 2C), proportion of ambiguous clustering [25] (PAC, Figure 2D) and average silhouette width (ASW, Figure 2E). According to Δ(K), the optimal cluster number was 5 because clustering stability increased for k=2 to 5 but almost not for k > 5 (Figure 2B, 2C). And according to PAC and ASW, we prefer to k=5 as the optimal cluster number in consideration of the major change before and after k=5, although the lowest PAC scores and the largest ASW values appeared at k=2 and 5 (Figure 2D, 2E). Therefore, the optimal cluster number was determined to be 5 and then 431 ccRCCs were classified into five robust clusters: R1 (n = 105, 24.4%), R2 (n=127, 29.5%), R3 (n = 83, 19.3%), R4 (n = 92, 21.3%) and R5 (n = 24, 5.6%) (Figure 2F and Supplementary Table S3).

**Figure 3:**
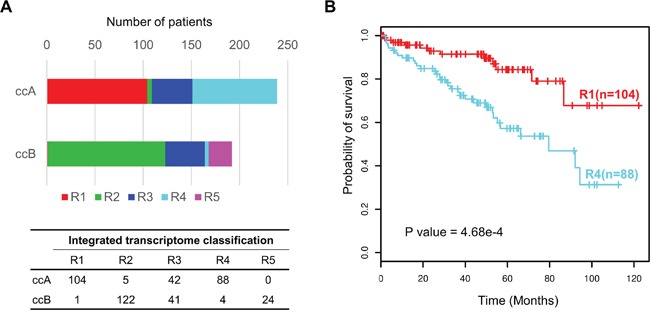
Prognostic value of integrated transcriptomic classification compared with ccA/ccB classification **A.** Overlap between our integrated transcriptomic classification and ccA/ccB clusters. The bottom panel shows the detailed crosstab table. **B.** Kaplan-Meier plot showing that clusters R1 and R4 can effectively separate the samples in ccA cluster in terms of overall survival (R1(n=104) vs R4 (n=88), log-rank test P = 4.68e-4).

The integrated transcriptomic classification shows high association with those classifications based on the single data type, such as ccA and ccB expression subtypes [21, 26] (*p*<1e-5), TCGA mRNA-based subtypes [11] (*p*<1e-5), TCGA miRNA-based subtypes [11] (*p*<1e-5) and the lncRNA-based subtypes [22] (*p*<1e-5) (Supplementary Table S4, Fishers' exact test). Interestingly, when compared to ccA and ccB subtypes, our clusters R1 and R4 divide ccA into two subgroups with significantly different survivals, clusters R2 and R5 correspond to ccB, and cluster R3 consists of almost equal numbers of ccA and ccB tumors which may account for roughly 20% unclassified tumors in the ccA/ccB classification scheme [21] (Figure 3A, 3B). The result suggests that our integrated transcriptomic classification may be a step forward and could divide ccRCCs into more elaborate subgroups.

### Somatic alteration analysis uncovers disparate characteristics of R5

**Figure 4:**
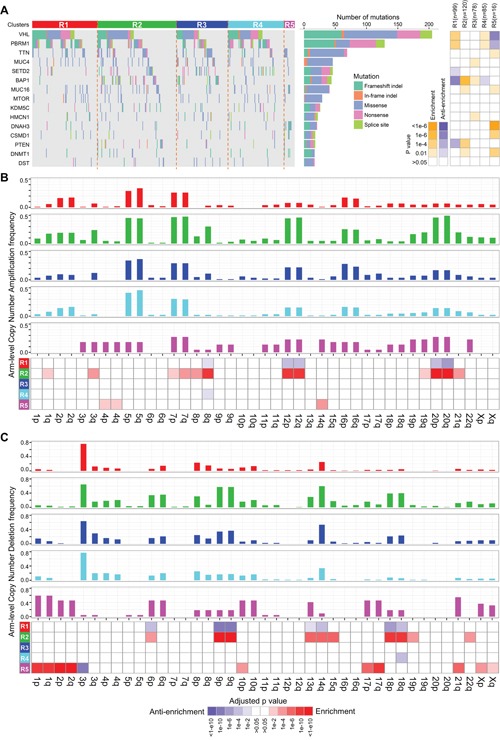
Distributions of somatic genomic alteration in five clusters identified by ICC **A.** Distributions of non-silent mutations of the top 15 most frequently mutated genes in ccRCC. The right panel represents significance of enrichment or anti-enrichment (one-sided Fisher's exact test) of non-silent mutation events for each gene within any particular cluster versus the other clusters. **B, C.** Distributions of arm-level copy number alterations (CNA), including amplification (B) and deletion (C), in five clusters. Bar plots of frequency of CNA in five clusters are on the top five panels. The bottom panel shows significance of enrichment or anti-enrichment (one-sided Fisher's exact test) of CNA events for each chromosome arms within any particular cluster versus the other clusters.

We next investigated the distributions of somatic alterations and observed different patterns among ccRCC clusters at gene mutation and CNA levels (Figure 4 and Supplementary Tables S5–S7). For example, mutations of *VHL* and *PBRM1* frequently occur in clusters R1, R2, R3 and R4 but scarcely in R5. *BAP1* mutations are abundant in R2 (20.83%) and R3 (17.95%) compared to R1 (1.01%) and R4 (4.71%) (*p*=5.86e-7, Fisher's exact test), whereas the *PBRM1* gene is mutated more frequently in R1 (44.44%) and R4 (40.00%) compared to R2 (26.67%) and R3 (24.36%) (*p*=1.65e-4) (Figure 4A and Supplementary Table S5). Moreover, R2 has more gains of chromosome 3q, 8q, 12, 20 (*p*<1e-4, Figure 4B and Supplementary Table S6) and losses of chromosome 6p, 9, 13q, 14q, 15q, 18 (*p*<1e-4, Figure 4C and Supplementary Table S7), showing the higher frequency of chromatin abnormality of R2.

Interestingly, cluster R5 has significantly disparate somatic alteration patterns compared to the other clusters. The well-known frequent somatic alterations in ccRCC, such as losses of chromosome 3p and gene mutations of *VHL*, *PBRM1* and *BAP1* [11], rarely occur in R5 (Supplementary Table S5). In contrast, R5 is significantly enriched for losses of chromosomes 1, 2, 6, 10p, 17, 21q, X (Figure 4C and Supplementary Table S7), which is a typical genetic characteristic of chRCC [6]. Interestingly, according to a re-examination of ccRCC tumor histology by pathologists from a recent report [27], 22 tumor samples in TCGA were misdiagnosed as ccRCCs and should have been documented to be a mixture of chRCC and ccpRCC. Among them, 20 tumors are involved in our study and 19 are included in cluster R5 (sensitivity = 95% (19/20), Supplementary Table S3). These results demonstrate that R5 should be a subclass of RCCs easily misdiagnosed as ccRCCs in clinic. Therefore, we excluded R5 from the integrated transcriptomic classification of ccRCCs.

### Clinicopathological characteristics and survival analysis of four ccRCC subtypes

**Figure 5:**
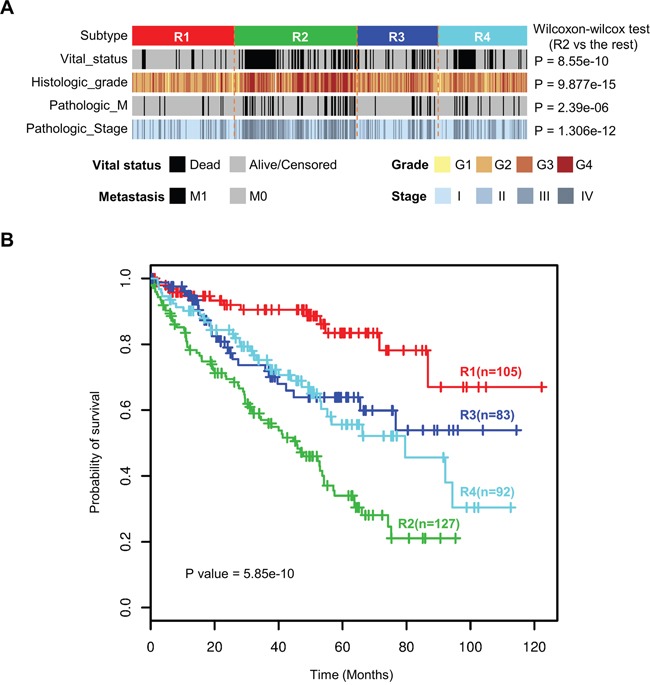
Four integrated transcriptomic subtypes were associated with different clinicopathological features and overall survival **A.** Subtype R2 was enriched for more distant metastasis and higher histologic grade and pathologic stage. **B.** Kaplan-Meier survival analysis revealed significantly different survival outcomes among the four integrated transcriptome subtypes.

We surveyed clinicopathological characteristics of four ccRCC subtypes (Table 1). Subtype R2 is enriched for more distant metastasis and higher histologic grade and pathologic stage, suggesting more aggressive behaviors of tumors in R2 (Figure 5A). Overall survival analysis reveals significantly different survival outcomes among the four integrated transcriptomic subtypes (Figure 5B). Furthermore, multivariate analysis demonstrates that our four integrated transcriptomic subtypes are independent prognostic factors after adjusting for clinical indices including gender, age, pathologic stage and histologic grade (Table 2).

**Table 1:** Clinical and pathological characteristics of integrated transcriptomic subtypes

		R1	R2	R3	R4	P value
Patients	Count	105	127	83	92	-
Age(in years)	Median	61	62	57	60	-
	Range	(29,86)	(26,85)	(32,88)	(38,88)	
Survival	Median survival time	Not reached	45.7	Not reached	79.5	-
	(in months)					
	5-year survival rate	83.40%	34%	63.90%	55.60%	
Gender	Female	51	35	21	40	0.00057*
	Male	54	92	62	52	
Histologic grade	G1	4	1	0	3	1.21E-13*
	G2	60	26	39	43	
	G3	37	53	36	40	
	G4	4	46	8	6	
Pathologic stage	I	67	27	44	46	2.35E-11**
	II	7	13	10	10	
	III	23	48	17	24	
	IV	8	39	12	12	
Tumor size	T1	67	29	47	46	3.82E-11**
	T2	9	17	13	12	
	T3	29	73	22	33	
	T4	0	8	1	1	
Lymph node status	N0	47	65	36	47	0.03599**
	N1	2	10	4	0	
Distant metastasis	M0	97	89	72	79	4.75E-05**
	M1	8	38	11	13	

* Fisher's exact test

** Kruskal-Wallis Rank Sum test

**Table 2:** Univariate and multivariate Cox analyses including the classification and clinical features

Variables	Ref	Univariate analysis		Multivariate analysis	
		H.R.(95% C.I.)	P value	H.R.(95% C.I.)	P value
Integrated Cluster	R1	-	1.25E-08	-	0.003402
	R2	5.581(3.087-10.090)	1.27E-08	2.984(1.589-5.603)	0.000673
	R3	2.441(1.242-4.796)	0.009608	2.393(1.212-4.722)	0.011903
	R4	2.949(1.560-5.576)	0.000876	3.072(1.621-5.823)	0.000582
Gender	Female	0.934(0.668-1.307)	0.692018	0.941(0.664-1.335)	0.735252
Age	-	1.033(1.018-1.047)	5.81E-06	1.033(1.017-1.049)	4.32E-05
Pathologic Stage	-	1.931(1.662-2.243)	1.00E-13	1.689(1.421-2.007)	2.66E-09
Histologic Grade	-	2.198(1.750-2.762)	1.37E-11	1.319(1.012-1.720)	0.040471

### Molecular expression patterns and pathway analysis

**Figure 6:**
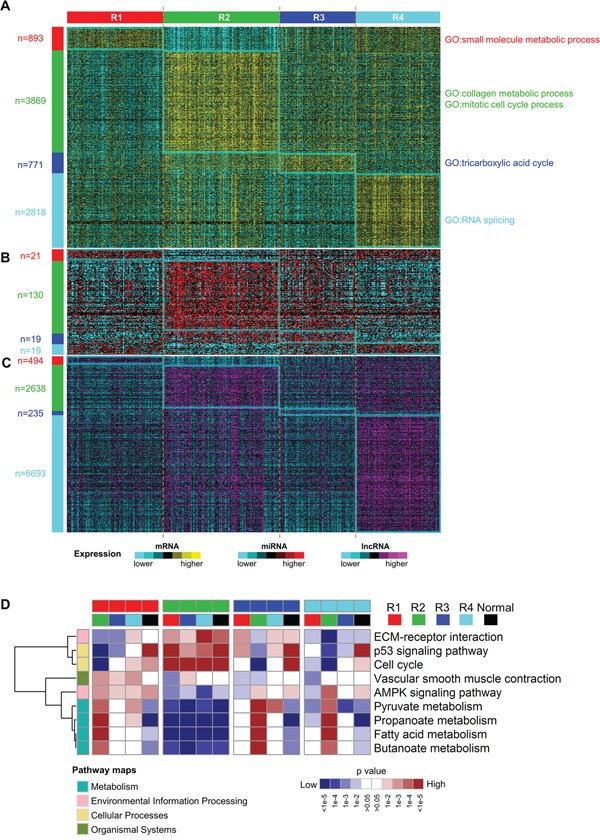
Different molecular expression patterns and pathways expression levels among subtypes **A-C.** subtype-specific mRNA (A), miRNA (B) and lncRNA (C) expression patterns among four ccRCC subtypes. The numbers of significantly highly expressed RNAs were labeled on the left. **D.** Heatmap of relative expression levels of selected pathways for all contrasts among ccRCC subtypes and normals by gene set analysis. The complete heatmap is shown in Supplementary Figure S3. Each subtype was compared with every other subtype and adjacent normal samples. These pairwise comparisons resulted in 16 columns and each column indicated which pathways were elevated or reduced when comparing the two subclasses indicated by the colors at the top of the heatmap. Categories of KEGG pathways were indicated by the colors at the left of the heatmap.

RNAs with subtype-specific expression may play important functions in individual molecular subtypes. So we identified the mRNAs, miRNAs and lncRNAs with significantly high expression (fold change > 1.5 and adjusted p value < 0.05) among four integrated transcriptomic subtypes by comparing each subtype with the rest using a moderated t-test [28] (Figure 6A–6C). Interestingly, the numbers of subtype-specific highly expressed mRNAs and lncRNAs in R2 and R4 are much larger than those in R1 and R3, implying that tumors in R2 and R4 are more active at transcriptomic level. In addition, highly expressed miRNAs in R2 are far more than those in other subtypes and highly expressed lncRNAs in R4 account for more than half (6693/12727=52.6%) of all the lncRNAs evaluated in this study. These results suggest that non-coding RNAs are important in ccRCC subtyping and that miRNAs and lncRNAs may play critical roles in R2 and R4, respectively.

To reveal biological functions related to each subtype, we performed gene ontology enrichment analysis on the top 500 overexpressed mRNAs using R package topGO [29] and then the top 50 most significantly enriched biological processes were summarized using the REVIGO webserver [30]. Four subtypes show distinct enriched biological processes (Figure 6A and Supplementary Figure S2). Consistent with previous reports [21, 31], upregulated metabolism-related genes, which are enriched in R1, correlate with good prognosis of ccRCC, and overexpression of genes involved in mitotic cell cycle, which are enriched in R2, are associated with worse prognosis. Moreover, collagen is the most abundant protein in the extracellular matrix (ECM). Upregulation of genes in collagen metabolic process may help cancer cells breach the ECM and escape, which may account for more invasive and metastatic features of tumors in R2 [32]. In addition, upregulated genes involved in RNA splicing in R4 implies that aberrant splicing events may contribute to disease progression of tumors in R4.

Next, we investigated the expression of pathways among four subtypes by pairwise comparisons of four subtypes and adjacent normal samples using gene set analysis [33] and KEGG pathway genes. Downregulated expression of various metabolic pathways are observed in all four ccRCC subtypes when compared to adjacent normal samples (Supplementary Figure S3), which is consistent with the central feature of oncologic-metabolic shift in ccRCC [11]. The widespread differentially expressed pathways are observed among the four ccRCC subtypes, which delineates the distinct pathway characteristics of subtypes. Notably, AMPK signaling pathway is more highly expressed in R1 compared to any other subtype. Activated AMPK signaling pathway can lead to inhibition of biosynthetic pathways and activation of catabolic pathways [34] and hence improve the oncologic-metabolic shift of ccRCC, which may explain good prognosis of R1. Subtype R2 has the highest expression of genes involved in cell cycle, p53 signaling pathway and ECM-receptor interaction and the lowest expression of genes involved in multiple metabolism pathways (Figure 6D), which may promote aggressive behavior of ccRCC and account for poor prognosis of R2.

### Triple-color co-expression network analysis

**Figure 7:**
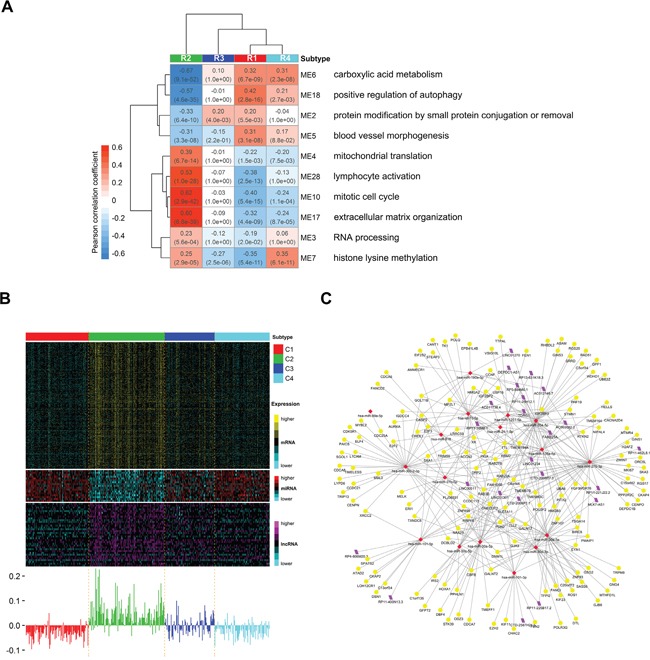
Distinct expression patterns of prognosis modules among subtypes and the potential regulatory network of miRNAs in module 10 **A.** Heatmap reporting correlations and corresponding p-values between prognosis modules and subtype phenotypes. Each module is represented by its module eigengene and the most significant biological processes for each module are shown on the right. **B.** Heatmap of genes belonging to prognosis module 10 (mitotic cell cyle) (top) and corresponding module eigengene values across samples (bottom). **C.** Visualization of the potential regulatory network of miRNAs in module 10. Yellow circle, red rhombus and purple rhomboid indicate mRNAs, miRNAs and lncRNAs, respectively. We predicted the targets (including mRNAs and lncRNAs) of miRNAs in module 10 using miRWalk2.0 webserver with default settings. Then those predicted targets included in module 10 were retained together with miRNAs for network visualization.

To systematically understand the potential regulation relationships among mRNAs, miRNAs and lncRNAs in ccRCC, we constructed a triple-color co-expression network of mRNAs, miRNAs and lncRNAs using weighted gene co-expression network analysis (WGCNA) [35] on a cohort of 407 ccRCCs included in R1-R4. Using unsupervised hierarchical clustering analysis and a dynamic hybrid tree cut algorithm, we identified 31 distinct triple-color co-expression modules (Supplementary Figure S4A, Supplementary Tables S8–S9). This result suggests that synergistic and regulatory effects among protein-coding and non-coding RNAs may exist widely in ccRCC and contribute to the identification of homogeneous integrated transcriptomic subtypes. In addition, we used the module eigengene (ME) to summarize expression of each module and to assess whether modules are associated with subtype phenotypes (See Methods). We observed significant expression differences in 28 modules among subtypes (Supplementary Figure S4B). We further identified 10 prognosis modules (M2-7, M10, M17-18, M28) (Figure 7A) with significant correlation with survival and enrichment of prognosis genes (See Methods). These results suggest that these triple-color modules, especially prognosis modules, may play important roles in tumorigenesis of ccRCC.

To explore biological functions of modules, we performed gene ontology enrichment analysis on mRNAs of each module using topGO [29]. Then we summarized the top 50 most significant biological processes using REVIGO [30] and defined the most significant summarized biological process as the function of the module. These module functions include diverse biological processes, such as mitotic cell cycle, carboxylic acid metabolism, blood vessel morphogenesis, RNA processing and extracellular matrix organization (Supplementary Figures S4B). Notably, among the 10 prognosis modules (Figure 7A), the functions of M5 (blood vessel morphogenesis), M6 (carboxylic acid metabolism) and M10 (mitotic cell cycle) have been reported to be critical in ccRCC [11]. Other prognosis modules such as M4 (mitochondrion translation), M7 (histone lysine methylation), M17 (extracellular matrix organization) and M18 (positive regulation of autophagy) may also exert important functions in tumorigenesis of ccRCC although they are less well-studied for the moment. Interestingly, the worst prognosis subtype R2 shows nearly completely opposite expression patterns of prognosis modules compared to the best prognosis subtype R1 (Figure 7A), suggesting that these prognosis modules together may characterize the molecular features of subtypes and correspond to their disparate survival outcomes. This result also shows that low expression of M6 (carboxylic acid metabolism) and M18 (positive regulation of autophagy) and high expression of M10 (mitotic cell cycle), M17 (extracellular matrix organization) and M28 (lymphocyte activation) may be associated with poor prognosis in ccRCC (absolute Pearson's correlation coefficient > 0.5 in R2). In addition, subtype R4 shows very close expression patterns with R1 except M7 (histone lysine methylation), which has significantly high expression in R4 but low expression in R1. The result implies that high expression of M7 (histone lysine methylation) may partially account for the worse prognosis of R4 when compared to R1.

Moreover, in prognosis modules, miRNAs and lncRNAs may play important regulation functions in associated biological processes. For example, in module M10 (mitotic cell cycle) (Figure 7B), miR-30a [36], let-7c [37, 38], miR-101 [39] and LINC00511 [40] (Figure 7C) have been reported to play regulation functions in tumor cell growth and proliferation. The rest miRNAs and lncRNAs in M10 may also participate in regulation of genes associated with cell cycle since they are highly co-expressed. Interestingly, DEPDC1, the antisense gene of lncRNA DEPDC1-AS1, plays a pivotal role in the regulation of mitotic progression [41]. DEPDC1 and DEPDC1-AS1 are both included in M10 and highly co-expressed. The result suggests that DEPDC1-AS1 may be involved in cell cycle as a cis-acting regulator of DEPDC1. Although detailed molecular mechanism need further research, our results still suggest that the prognosis modules may represent critical functional modules in ccRCC and those miRNAs and lncRNAs in modules may play important regulation functions in associated biological processes.

## DISCUSSION

In this study, we introduced ICC to integrate mRNA, miRNA and lncRNA expression profiles for ccRCC subtyping. We identified one RCC subclass easily misdiagnosed as ccRCCs in clinic and four robust ccRCC subtypes that were associated with different clinicopathologic features, genomic aberrations and molecular expression patterns. Survival analysis showed that our classification could separate ccRCC tumors with different overall survivals. Functional analysis of four ccRCC subtypes characterized distinct features of biological processes and pathways. In addition, triple-color co-expression network analysis depicted co-expression relationships among mRNAs, miRNAs and lncRNAs in ccRCC and identified 10 prognosis modules characterizing molecular features of four subtypes. Our results show that the integrative analysis of protein-coding genes and non-coding RNAs may contribute to delineate molecular heterogeneity within ccRCC and may help guiding treatment of ccRCC.

Our ICC approach integrates multiple data types by transforming the information of each data type into a PSM and merging PSMs into an fPSM. The final clustering result is determined by consensus clustering on fPSM. One advantage of the method is that it does not require normalization of data across multiple types or platforms prior to integrating them. Any data type can be integrated into analysis, including gene mutations, CNA, DNA methylation and gene/miRNA/protein expression profiles. We used ICC to integrate mRNA, miRNA and lncRNA expression profiles of ccRCCs and successfully identified five robust clusters including one RCC subclass misdiagnosed as ccRCCs and four clinically relevant subtypes. This integrative analysis framework can also be applied to other cancer types.

Among the five clusters identified with ICC, cluster R5 shows significantly different somatic alterations patterns with other clusters. A recent report shows that 22 ccRCC samples in TCGA are misdiagnosed as ccRCCs and should be a mixture of chRCC and ccpRCC [27]. Cluster R5 accurately recognized 19 out of 20 misdiagnosed samples included in our study (True Positive Rate (TPR) = 86.4%, higher than TPR = 64.5% by lncRNA-based classification [22]). The result demonstrates the ability of integrative analysis of protein coding genes and non-coding RNAs to distinguish other RCCs from ccRCCs.

Our integrated transcriptomic classification identified four robust ccRCC subtypes. When compared to previously established gene expression ccA/ccB classification, our subtyping system can further divide ccA into two subgroups R1 and R4 with significantly different survival. Moreover, more than half (52.6%) of lncRNAs are significantly highly expressed in R4, much more than those in other subtypes, which makes R4 a potential lncRNA-dependent subtype. These results suggest that integrating information from both protein coding and non-coding RNAs may contribute to capturing the heterogeneity of ccRCC and could help dividing ccRCCs into more elaborate subgroups.

To our knowledge, this is the first integrative analysis of protein-coding genes and non-coding RNAs in cancer subtyping. Our four integrated transcriptomic subtypes significantly correlate with clinicopathologic and molecular features. Although this result should be verified in a larger sample size from multiple centers, our study demonstrates that integrative analysis of protein-coding genes and non-coding RNAs are valuable for ccRCC subtyping.

## MATERIALS AND METHODS

### The cancer genome atlas (TCGA) data

All ccRCC patients are from the kidney renal clear cell carcinoma (KIRC) cohort of The Cancer Genome Atlas (TCGA) project [11]. Multi-omics datasets of a cohort of 431 ccRCC patients with matched mRNA, miRNA and lncRNA expression profiles were used in this study (Supplementary Tables S2–S3). Level 3 mRNA-seq RSEM data (n = 533) and Level 3 miRNA-seq RPM data (n = 516) were obtained from the Broad Institute GDAC FireBrowse (TCGA data version 20150601, http://firebrowse.org/). LncRNA RPKM data (n = 448) was downloaded from TANRIC database [42]. Gene mutation and copy number alteration data were archived from Mutation MutSig2.0 Analyses results and CopyNumber Gistic2 Analyses results on the Broad Institute GDAC FireBrowse (TCGA data version 20150401). Clinical information data were downloaded from UCSC cancer browser [43].

### Transcriptome data preprocessing

The same preprocessing flow was applied to mRNA, miRNA and lncRNA datasets independently. Given a dataset, the RNA expression matrix was obtained by substituting the smallest non-zero value for all zero or NA values in the dataset and taking a base-2 logarithmic transformation. Before clustering, RNA expression matrix was median centered by RNA and the most variable RNAs was selected using median absolute deviation. Finally, the most variable 2,500 mRNAs, 1,500 lncRNAs and 300 miRNAs were used for the unsupervised clustering analysis.

### Integration of multiple transcriptome data types using consensus clustering

For the first step of the ICC method, we employed consensus clustering algorithm [24] to integrate multiple transcriptome data types, including mRNA expression, miRNA expression and lncRNA expression profiles. First, we constructed the patient similarity matrix (PSM) represented by a consensus matrix for each data type independently (Figure 1A, 1B). Due to the comparability of the consensus matrix, we then combined three PSMs into a fused PSM (fPSM) by matrix addition (Figure 1C). Specifically, we initially performed consensus clustering on each filtered RNA expression matrix to construct the consensus matrix using the R package ConsensusClusterPlus. To ensure comparability of clustering results based on different data types, the same clustering parameters were used for different data types and different cluster numbers. For any given data type and cluster number k, we conducted 1,000 runs (reps) of agglomerative hierarchical clustering algorithm (clusterAlg) using the resampled data with 80% patient resampling (pItem) and 80% RNA resampling (pFeature). The distance measurement was set as Pearson correlation (distance) and linkage function was set as “ward.D” (both innerLinkage and finalLinkage). Then, a single combined consensus matrix was achieved by summation of three consensus matrices derived from different data types. We consider the combined consensus matrix as a fPSM capturing a combination of multiple patient similarity measures derived from different transcriptome data types.

### Assessment of clustering stability

Given multiple transcriptome data sets and a specific cluster number k, we can now achieve a fPSM for clustering. Furthermore, to obtain stable clustering results, we used consensus clustering to the fPSM to assess clustering stability (Figure 1D). Considering the specific properties of the PSM (patients × patients), we used a custom implementation of consensus clustering to resample patients identically from rows and columns of the fPSM. We carried out 1,000 runs of “ward.D” linkage hierarchical clustering using the resampled data with 80% patient resampling and 1-Spearman correlation as distance. Then a final consensus matrix was calculated. Finally, we performed hierarchical clustering to achieve the cluster membership result with 1-the final consensus matrix as a distance matrix and “ward.D” as the linkage function.

### Selection of optimal cluster number

We selected a maximum cluster number k of 12 for assessment of optimal cluster number. To intuitively inspect clustering results, we used the R package pheatmap for visualization of the final consensus matrix for each cluster number. We also calculated empirical cumulative distribution (CDF) for the final consensus matrix and used the proportional area change under CDF (Δ(k)) for selection of the optimal k. According to the Δ(k) vs k plot, the optimal k is the k for which the Δ(k) value will be close to zero. To avoid a subjective judgement, we also investigated the proportion of ambiguous clustering (PAC) score and average silhouette width for each cluster number k, and the k for which the lowest PAC score or highest average silhouette width (ASW) appears can be considered to be the optimal K. The optimal number of clusters was determined by a combination of Δ(K), PAC and ASW.

### Gene-set analysis

We performed gene-set analysis among ccRCC subtypes and adjacent normal sample group using the R package PIANO [33] and KEGG pathway genes set from NCBI BioSystems [44]. The *p*-value for each gene calculated for differential expression analysis was used as gene-wise statistics. Fisher's combined probability test was used to aggregate data into a gene-set *p*-value. The “geneSampling” method was used for significance assessment of gene sets. The significant up(/down)-regulated pathways were filtered and only the dominantly up- or down-regulated pathways were reported.

### Weighted gene co-expression network analysis

A signed weighted co-expression network was constructed using the R package WGCNA [35]. mRNAs, miRNAs and lncRNAs expressed in more than 70% samples of 407 ccRCCs (patients in R1-R4) were independently filtered. Then 27,543 genes (18,288 mRNAs, 566 miRNAs and 8,689 lncRNAs) were included for network construction. In order to seize the negative regulation functions of miRNAs, we multiplied miRNA expression values by −1 so that miRNAs could be positive correlation with their potential targets and clustered together in the signed network. First, a matrix of pairwise correlations between all pairs of RNAs across 407 ccRCC samples was calculated. Then, an adjacency matrix was computed by raising the 0.5*(1+correlation matrix) to the power of 6, which is the suggested value for more than 60 samples in a signed hybrid network. Based on the adjacency matrix, a topological overlap matrix was constructed. Using 1-topological overlap matrix as a dissimilarity matrix, the average linkage hierarchical clustering was performed. Finally, robustly defined modules were identified by cutting the hierarchical clustering tree using the dynamic hybrid tree cut algorithm. We summarized each module by the module eigengene (ME) represented by the first principal component of the scaled module expression profiles, which explains the major variation of module expression. Expression values of MEs were used for assessing correlations of modules with survival and five subtype phenotypes. Here, the subtype phenotype was introduced as a binary trait variable across all samples. In this case, phenotypic trait is the subtype category, that is R1-R4. To define prognosis modules, we also identified 2,822 prognosis genes (2,494 mRNAs, 27 miRNAs and 301 lncRNAs) (Supplementary Table S9) which were significantly correlated with survival after adjusting by FDR among all 27,543 genes (Adjusted p value < 1e-4).

### Statistical analysis

All statistical analysis was performed using R language [45]. The R package survival was used for survival analysis. Kaplan-Meier methods and a log-rank test were used to assess differences between survival distributions. Univariate and multivariate models were calculated using Cox proportional hazards regression. The R package limma [28] was employed for differential expression analysis. A Fisher's exact test was used to compute *p*-values from a contingency table. The Benjamini and Hochberg method was used for multiple testing adjustment and 0.05 was considered statistically significant, unless otherwise noted.

## SUPPLEMENTARY FIGURES AND TABLES




