# Supplementary material for: Integrative analysis of protein-coding and non-coding RNAs identifies clinically relevant subtypes of clear cell renal cell carcinoma
Source: Oncotarget. 2016 Sep 29;7(50):82671–85. doi: 10.18632/oncotarget.12340 (PMC5347723; doi:10.18632/oncotarget.12340)
Supplement: Supplementary file 1 [file oncotarget-07-82671-s001.pdf]

Integrative analysis of protein-coding and non-coding RNAs identifies clinically relevant subtypes of clear cell renal cell carcinoma

SUPPLEMENTARY FIGURES AND TABLES

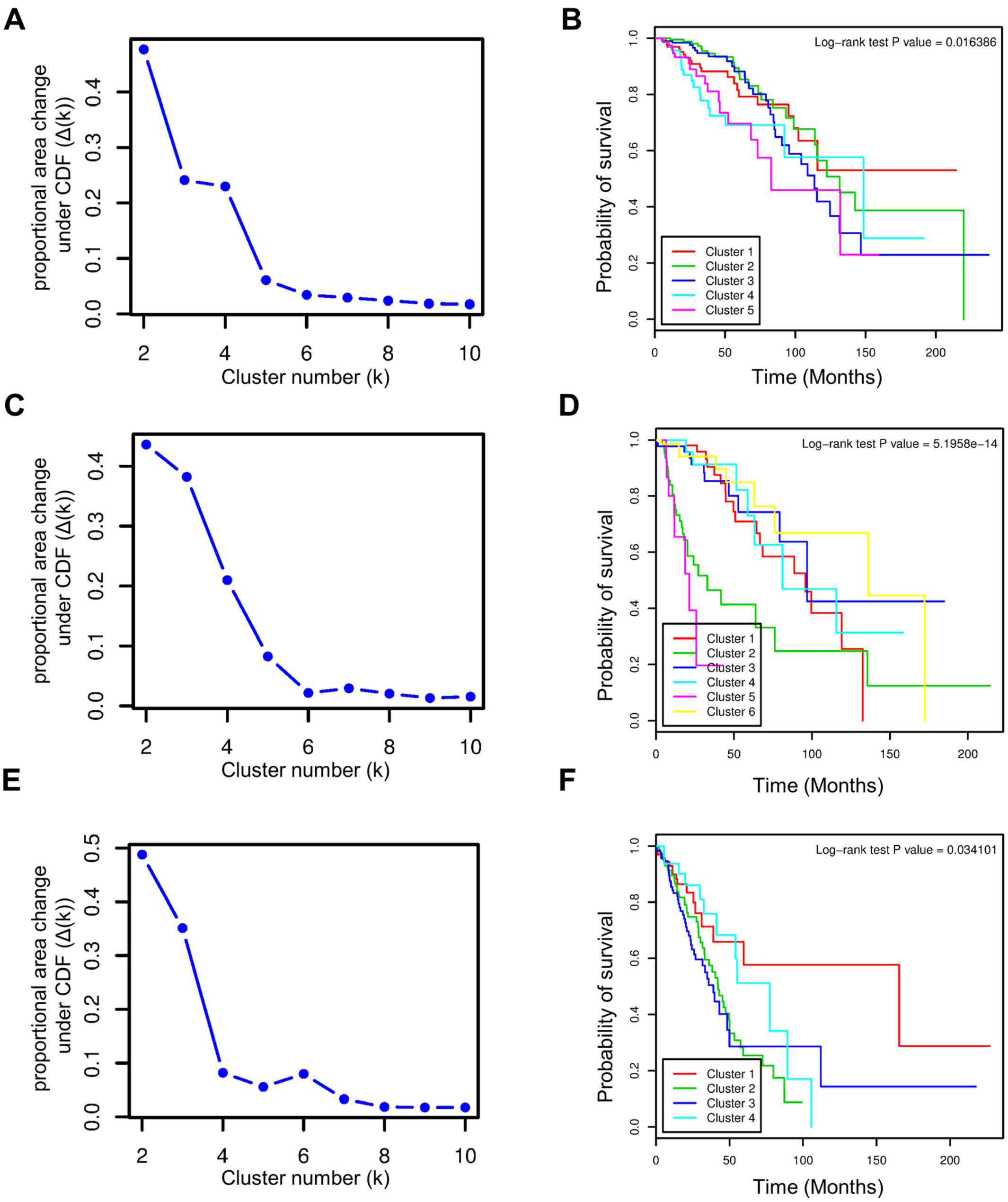

Supplementary Figure S1: Validation of the feasibility of ICC using overall survival analysis on three TCGA cohorts. The Δ(k) vs k plots indicate the optimal cluster number where the 'elbow' occurs, that is k = 5 for BRCA **A.** k = 6 for LGG **C.** and k = 4 for LUAD **E.** Given the optimal clustering, significant survival differences were observed among subgroups of BRCA **B.** LGG **D.** and LUAD **F.** respectively.

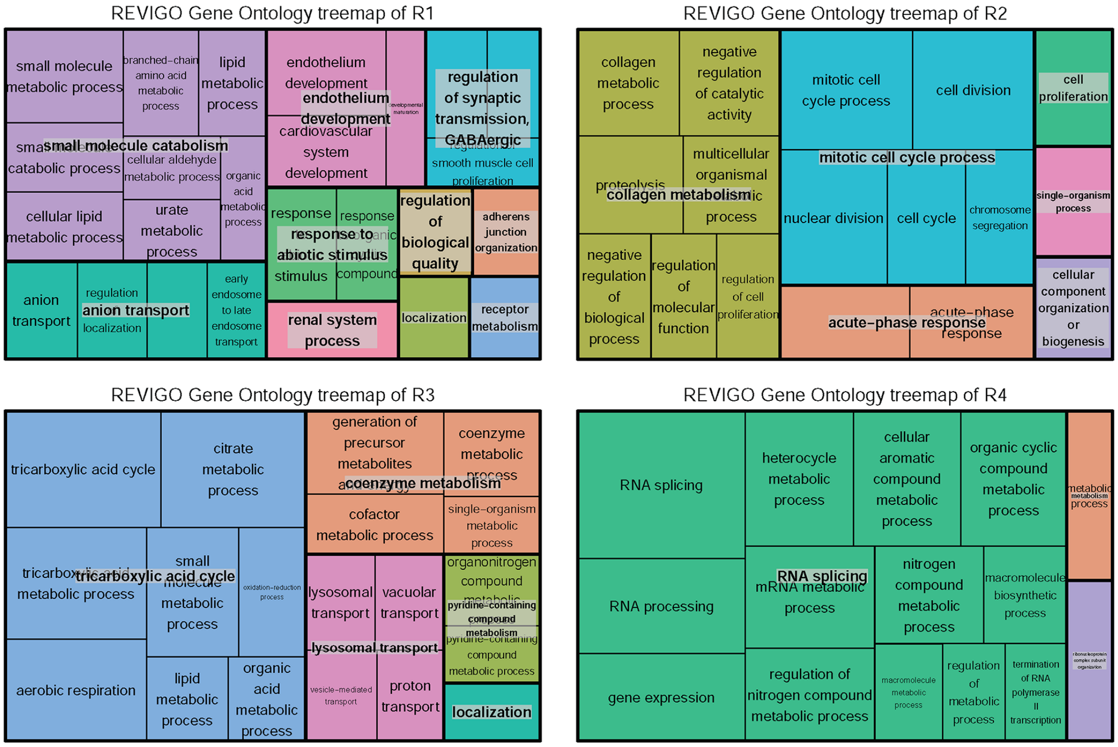

Supplementary Figure S2: Visualization of GO:BP terms significantly enriched for subtype-specific highly expressed genes.

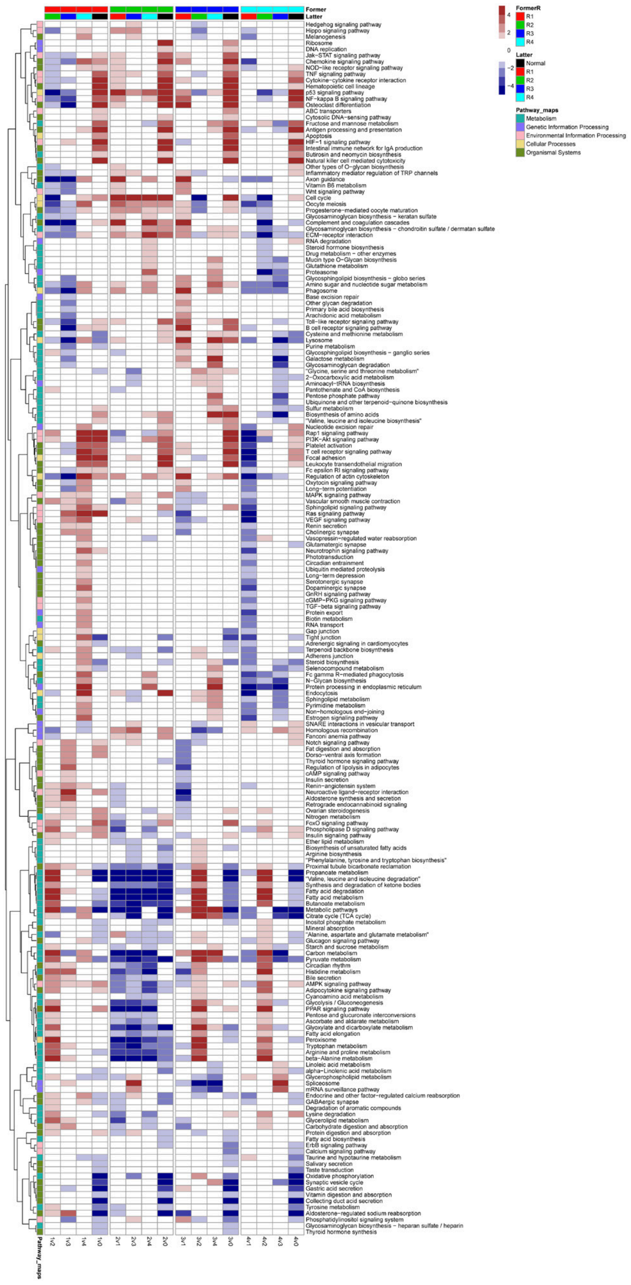

Supplementary Figure S3: Heatmap of relative pathway expression levels for all contrasts among ccRCC subtypes and normals by gene set analysis. Each subtype was compared with every other subtype and adjacent normal samples. These pairwise comparisons resulted in 16 columns and each column indicated which pathways were elevated or reduced when comparing the two subclasses indicated by the colors at the top of the heatmap. Categories of KEGG pathways indicated by the colors at the left of the heatmap. This data shows different pathway expression patterns among subtypes.

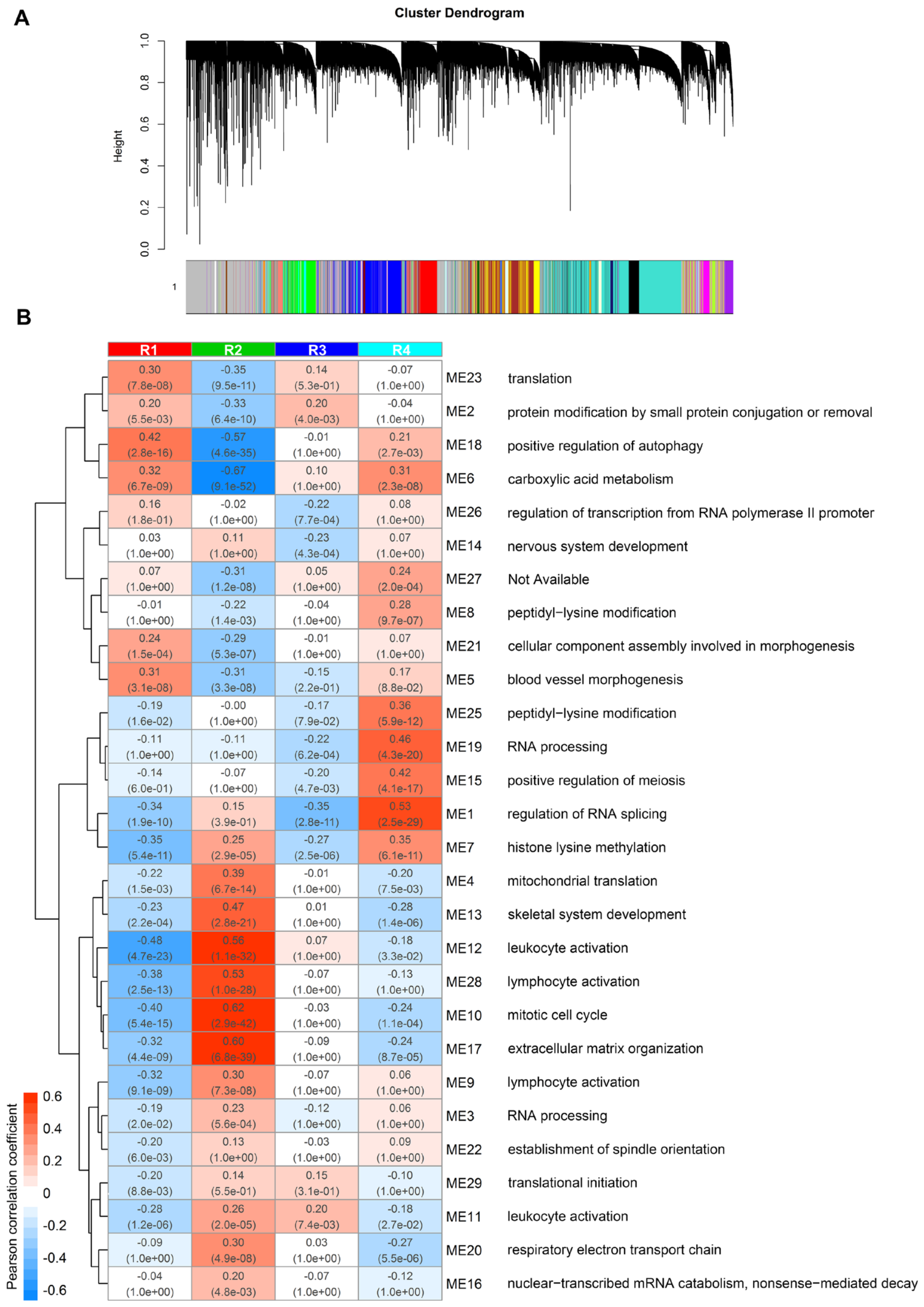

Supplementary Figure S4: Triple-color co-expression network analysis identified 31 modules of which 28 modules were differentially expressed among subtypes. A. Hierarchical cluster tree showing co-expression modules identified using WGCNA and modules are labelled by colors. **B.** Heatmap reporting correlations and corresponding p-values between modules and subtype phenotypes. Each module is represented by its module eigengene. The most significant biological process for each module was shown on the right.

Supplementary Table S1. Summary of the three TCGA cohorts used for validation

Cohorts	Data Type	Normalization	Cases	Cases in this study	Data source
BRCA	mRNA expression	RSEM	1091	819	Firebrowse
	miRNA expression	RPM	1078	819	Firebrowse
	lncRNA expression	RPKM	837	819	TANRIC
	Clinical Information	-	1241	819	UCSC Cancer Browser
LGG	mRNA expression	RSEM	513	482	Firebrowse
	miRNA expression	RPM	512	482	Firebrowse
	lncRNA expression	RPKM	486	482	TANRIC
	Clinical Information	-	530	482	UCSC Cancer Browser
LUAD	mRNA expression	RSEM	488	483	Firebrowse
	miRNA expression	RPM	513	483	Firebrowse
	lncRNA expression	RPKM	488	483	TANRIC
	Clinical Information	-	662	483	UCSC Cancer Browser

Supplementary Table S2: Summary of TCGA-KIRC datasets used in this study

Data Type	Normalization	Cases	Cases in this study	Data source
Mutation	-	451	398	Firebrowse
Copy Number	-	528	426	Firebrowse
mRNA expression	RSEM	533	431	Firebrowse
miRNA expression	RPM	516	431	Firebrowse
lncRNA expression	RPKM	448	431	TANRIC
Clinical Information	-	528	431	UCSC Cancer Browser

Supplementary Table S3: Clinical information and Integrated transcriptomic classification assignment of the 431 ccRCCs used in this study

See Supplementary File 1

Supplementary Table S4: Integrated transcriptomic classification identified by ICC show high association with other established ccRCC classifications

		Integrated transcriptomic classification					Fisher's Exact test
		**R1**	**R2**	**R3**	**R4**	**R5**	
microarray expression	ccA	104	5	42	88	0	<1e-5
	ccB	1	122	41	4	24	
TCGA mRNA-based	m1	58	0	29	39	0	<1e-5
	m2	5	30	5	40	0	
	m3	4	61	20	0	4	
	m4	25	25	21	10	0	
TCGA miRNA-based	mi1	26	6	12	26	4	<1e-5
	mi2	0	78	21	3	0	
	mi3	46	9	28	50	0	
	mi4	25	16	15	7	0	
lncRNA-based	C1	19	8	5	88	0	<1e-5
	C2	0	99	5	1	1	
	C3	80	19	62	1	0	
	C4	1	0	10	0	23	

Supplementary Table S5: Gene mutation profiles among five clusters

See Supplementary File 1

Supplementary Table S6: Chromosomal arm-level amplifications among five clusters

See Supplementary File 1

Supplementary Table S7: Chromosomal arm-level deletions among five clusters

See Supplementary File 1

Supplementary Table S8: Module-wise statistics of the triple-color co-expression network

See Supplementary File 1

Supplementary Table S9: Gene-wise statistics of the triple-color co-expression network

See Supplementary File 1
